# Three-stage niobium mineralization at Bayan Obo, China

**DOI:** 10.1093/nsr/nwae063

**Published:** 2024-02-22

**Authors:** Yan Yu, Yang Li, Yu Liu, Xiao-Xiao Ling, Li-Guang Wu, Li Yang, Lan Yang, Bo Yang, Yong-Gang Zhao, Xian-Hua Li

**Affiliations:** State Key Laboratory of Lithospheric Evolution, Institute of Geology and Geophysics, Chinese Academy of Sciences, Beijing 100029, China; College of Earth and Planetary Science, University of Chinese Academy of Sciences, Beijing 100049, China; Ministry of Education Key Laboratory of Orogenic Belts and Crustal Evolution, School of Earth and Space Sciences, Peking University, Beijing 100871, China; State Key Laboratory of Lithospheric Evolution, Institute of Geology and Geophysics, Chinese Academy of Sciences, Beijing 100029, China; State Key Laboratory of Lithospheric Evolution, Institute of Geology and Geophysics, Chinese Academy of Sciences, Beijing 100029, China; State Key Laboratory of Lithospheric Evolution, Institute of Geology and Geophysics, Chinese Academy of Sciences, Beijing 100029, China; Mining Research Institute, Baotou Iron and Steel (Group) Co., LTD, Baotou 014010, China; State Key Laboratory of Lithospheric Evolution, Institute of Geology and Geophysics, Chinese Academy of Sciences, Beijing 100029, China; College of Earth and Planetary Science, University of Chinese Academy of Sciences, Beijing 100049, China; State Key Laboratory of Lithospheric Evolution, Institute of Geology and Geophysics, Chinese Academy of Sciences, Beijing 100029, China; College of Earth and Planetary Science, University of Chinese Academy of Sciences, Beijing 100049, China; Mining Research Institute, Baotou Iron and Steel (Group) Co., LTD, Baotou 014010, China; Mining Research Institute, Baotou Iron and Steel (Group) Co., LTD, Baotou 014010, China; State Key Laboratory of Lithospheric Evolution, Institute of Geology and Geophysics, Chinese Academy of Sciences, Beijing 100029, China; College of Earth and Planetary Science, University of Chinese Academy of Sciences, Beijing 100049, China; State Key Laboratory of Baiyunobo Rare Earth Resource Research and Comprehensive Utilization, Baotou 014000, China

**Keywords:** niobium mineralization, U-Pb dating, ferrocolumbite, Bayan Obo

## Abstract

The Chinese Bayan Obo deposit is a world-class rare earth element (REE) deposit with considerable niobium (Nb) and iron (Fe) resources. A complete genetic understanding on all metals is fundamental for establishing genetic models at Bayan Obo. With extensive research being focused on REE enrichment, the timing and controls of Nb enrichment remain unresolved at Bayan Obo, which is mainly due to the challenges in dating, i.e. multistage thermal events, fine-grained minerals with complex textures and the rare occurrence of uranium-enriched minerals with mature dating methods. Based on robust geological and petrographic frameworks, here we conducted ion probe uranium-lead (U-Pb) dating of ferrocolumbite to unravel the timing, hence the genesis of Nb mineralization. Three types of hydrothermal ferrocolumbites—key Nb-bearing minerals—are identified based on their textures and mineral assemblages. They yield U-Pb ages of 1312 ± 47 Ma (*n* = 99), 438 ± 7 Ma (*n* = 93), and 268 ± 5 Ma (*n* = 19), respectively. In line with deposit geology, we tentatively link the first, second and third stage Nb mineralization to Mesoproterozoic carbonatite magmatism, ubiquitous early Paleozoic hydrothermal activity, and Permian granitic magmatism, respectively. While quantifying the contribution of metal endowment from each stage requires further investigation, our new dates highlight that multi-stage mineralization is critical for Nb enrichment at Bayan Obo, which may also have implications for the enrichment mechanism of Nb in REE deposits in general.

## Teaser

Robust geological framework and accurate geochronology reveal three stages of Nb mineralization at Bayan Obo, and highlight the critical role of multi-stage process for the formation of world-class ore deposits.

## INTRODUCTION

Carbonatite-related mineralization is the world's primary source of rare earth element (REE) and niobium (Nb), with the overall metal endowment being controlled by a few deposits. For example, the Araxá and Catalão-II (Brazil) and St. Honoré (Canada) deposits account for ∼98% of annual global Nb production, while the Bayan Obo (China) deposit accounts for ∼40% of annual global REE production [1]. Despite their economic importance and extensive studies, processes controlling metal enrichment in these giant systems remain controversial. The Bayan Obo deposit, renowned for its vast REE resources and substantial Nb and iron (Fe) reserves, is a classic example for this puzzle.

Despite 70 years of study (e.g. [2,3]), the timing of mineralization at Bayan Obo remains debated. Based on thorium-lead (Th-Pb) isochron dating of monazite and bastnäsite, the two principle REE minerals, Bayan Obo was proposed as a ∼0.4 Ga hydrothermal deposit related to Paleo-Asian Oceanic plate subduction [4,5]. In contrast, samarium-neodymium (Sm-Nd) isochron dating of REE minerals predominately defines an errorchron at ∼1.3 Ga [6]. In line with ∼1.3 Ga zircon Th-Pb dates from carbonatite dykes [7,8], a Mesoproterozoic carbonatite origin for Bayan Obo was proposed. Further Th-Pb dating of monazite and bastnäsite using LA-ICP-MS yield disparate dates ranging from ∼1.2 Ga to 0.26 Ga, with two peaks at ∼0.4 Ga and ∼0.27 Ga [9]. These disparate dates either have been used to argue for a protracted REE mineralization over ∼1 billion years [10], or remobilizing existing ∼1.3 Ga mineralization at ∼0.4 Ga and ∼0.27 Ga [9].

Existing dates of Nb mineralization are scarce, based on the occurrence of pyrochlore [(Ca, Na)_2_Nb_2_O_6_(OH, F)] from skarn in contact with Permian granites [5], and a Th-Pb isochron date at ∼273 Ma (*n* = 7;) for aeschynites [Ce(Ti, Nb)_2_O_6_] [11], a Permian stage Nb mineralization was suggested. In contrast, aeschynites in vein-type ores yielded disparate results from 658 ± 36 Ma (Sm-Nd isochron, *n* = 5; [12]) to 438 ± 25 Ma (Th-Pb isochron, *n* = 4; [5]), to 290 ± 15 Ma (Rb-Sr isochron, *n* = 4; [12]). Variability in these dates from the same ore type may result from isotopic system disturbance [12].

Deciphering the genesis of Bayan Obo relies on faithful interpretation of radiometric dates. The REE- and Th-rich nature of minerals like monazite and bastnäsite has prompted the widespread use of Sm-Nd and Th-Pb dating. However, the limited range of Sm/Nd ratios poses challenges for accurate and precise isochron dating, evident in substantial errors tied to errorchron [13]. Meanwhile, Th-Pb dating has yielded dates ranging from ∼1.2 to 0.26 Ga [9]. Given the common occurrence of galena and the inability of common lead corrections using LA-ICP-MS, the variations in Th-Pb dating could be partly explained by varying proportions of common lead. Additionally, this variability may also be linked to multiple thermal events, which caused the formation of multistage minerals and open system behavior of the Th-Pb system [14].

High-quality radiometric dating is essential to advance our understanding on the genesis of Bayan Obo. Here we approach this challenge from Nb mineralization and focus on dating ferrocolumbite [(Fe, Mn) (Nb, Ti, Ta)_2_O_6_]. As a major Nb-bearing mineral at Bayan Obo [15,16], ferrocolumbite constitutes ∼90% Nb in the dolomite host rock [17]. They incorporate significant amounts of U (up to hundreds of ppm), and leverage the dual U decay system (^238^U-^206^Pb and ^235^U-^207^Pb) as a powerful tool for dating complex geological systems and evaluating closed-system behavior.

Here we conducted a high-spatial-resolution (∼10 × 15 μm^2^) secondary ion mass spectroscopy (SIMS) ferrocolumbite U-Pb dating approach using a matrix-effect correction strategy [18]. We successfully obtained the first set of U-Pb ages for Nb mineralization at Bayan Obo, which were used to yield implications for deposit genesis.

## DEPOSIT GEOLOGY AND SAMPLES

**Figure 1.**
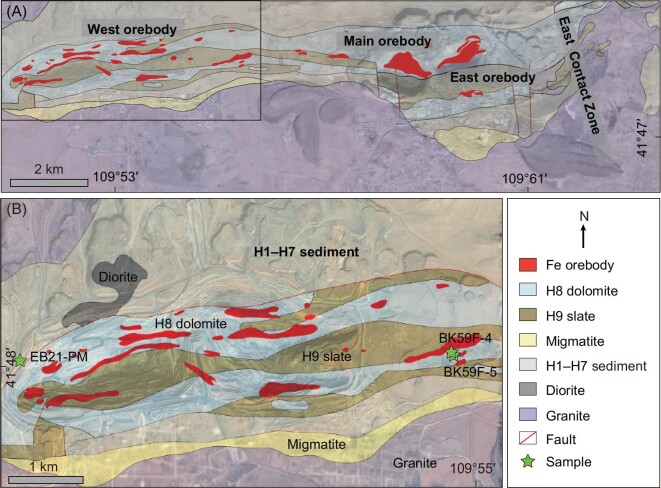
(A) Geological map of the Bayan Obo deposit and surrounding area. (B) Simplified geological map of the West orebody. The green star shows the locations of studied samples. The geological map was modified after [48,49].

The Bayan Obo deposit is hosted primarily by a dolomitic unit within the Mesoproterozoic Bayan Obo Group (H1–H9 units; Fig. 1A; [5]). The dolomitic unit was initially referred as ‘H8 dolomite’ [5], which is now considered to be carbonatite [19–23]. Three crucial tectono-thermal events influenced the Bayan Obo deposit, including Mesoproterozoic carbonatite magmatism, early Paleozoic hydrothermal alteration event, and Permian granitic magmatism. Abundant carbonatite dykes adjacent to the deposit [8,24], of which a few have been dated at ∼1.4–1.2 Ga by zircon U-Th-Pb dating [7,8], pointing to a Mesoproterozoic carbonatite magmatism. Undeformed to weakly deformed veinlets are commonly observed cutting across both the orebody and the H8 dolomite [25]. These veinlets exhibit diverse mineral compositions, encompassing REE- and Nb-bearing minerals, along with gangue minerals like fluorite, aegirine, alkaline amphibole, mica, pyrite, baryte, and molybdenite. Various dating results, including Sm-Nd, Rb-Sr, Re-Os isochron ages and Th-Pb dates, suggest that these veins were formed at ∼0.4–0.5 Ga [21,25–27]. Extensive Permian granites border the deposit to the south and east, leading to extensive hydrothermal metasomatism at the contact zone between granites and H8 dolomite in the east mining area—the so called East Contact Zone [5,28,29].

**Figure 2.**
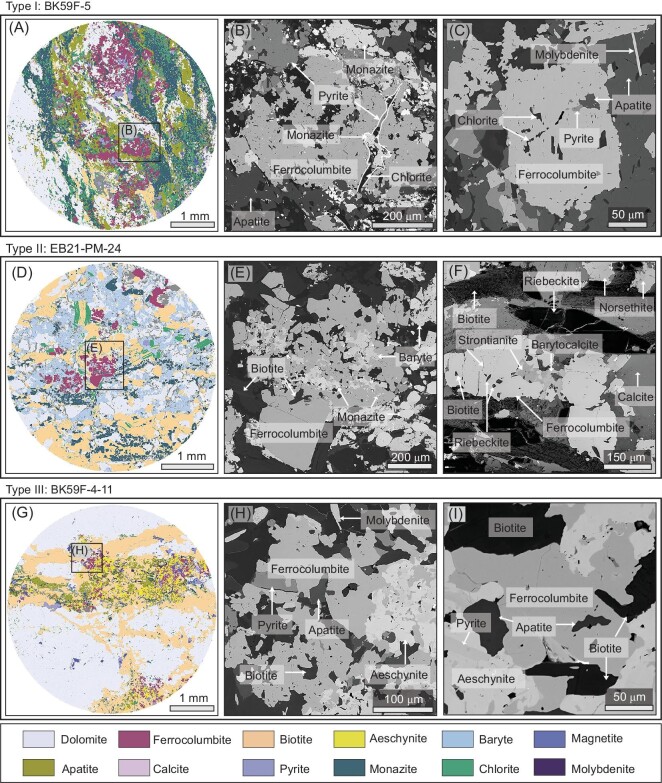
TEMSCAN TIMA mineral mapping and backscattered electron images of ferrocolumbite samples from the West orebody, Bayan Obo. (A–C) Images of the sample with type I ferrocolumbite. The ferrocolumbite coexists with and includes hydrothermal minerals. (D–F) Images of the sample with type II ferrocolumbite. The ferrocolumbite has inclusions of baryte, monazite, biotite, riebeckite and strontianite. (G–I) Images of the sample with type III ferrocolumbite. Ferrocolumbite is associated with aeschynite, biotite, apatite, monazite, pyrite, and molybdenite.

The Bayan Obo deposit comprises West, Main, and East orebodies (Fig. 1A), with Nb mineralization being particularly enriched in the West orebody and East Contact Zone [5]. The Main and East orebody each consists of a single lenticular-shaped orebody, whereas the West orebody is composed of several small orebodies (Fig. 1B, [2,5]). Within this deposit, monazite and REE fluorocarbonates are the major host of REE, while Fe resource is mainly found in magnetite and hematite, and Nb resource is primarily hosted by aeschynite, ferrocolumbite, fergusonite, pyrochlore, ilmenorutile, and baotite [15]. Through extensive field observations, representative samples were collected from outcrops of the open pit and drill cores. Based on the mineral occurrences of ferrocolumbites and their paragenetic mineral association of hydrothermal alteration in the host dolomite, three types of ferrocolumbite were identified.

All the three types of ferrocolumbite are hosted in dolomitic rocks, which were named dolomitic-type ores [11], but their alteration degrees, petrography textures, and mineral assemblages are distinct. Type Ⅰ ferrocolumbite is characterized by intense metasomatism. The original dolomite protolith (∼40 vol.%) experienced hydrothermal overprint with veins comprising of apatite (∼25 vol.%), chlorite (∼10 vol.%), biotite (∼8 vol.%), and monazite (∼10 vol.%, Fig. 2A–C). Type Ⅰ ferrocolumbite (∼5 vol.%, 10–400 μm) mostly occurs as subhedral to anhedral grains within the interstices of dolomite (Fig. 2A). Many ferrocolumbite grains have been sliced into incomplete remnants with curved serrated edges, and contain numerous fractures and inclusions of pyrite, chlorite, apatite, and monazite (Fig. 2B and C), while some grains still retain their original columnar shapes (Fig. 2C). Type Ⅱ ferrocolumbite (∼3 vol.%, 20–500 μm) is anhedral to subhedral and is distinctly characterized by association with mineral assemblages rich in Sr, Ba, and alkali (Fig. 2E and F). Their host protolith (∼55 vol.%, dolomite and calcite) is enriched in Sr and Ba, as evidenced by Sr- and Ba-rich minerals (∼10 vol.%, norsethite, strontianite, and barytocalcite). Their common association with alkaline-rich minerals (∼15 vol.%, biotite and riebeckite, Fig. 2D–F), and hosting abundant mineral inclusions (biotite, riebeckite, baryte, and monazite, Fig. 2E and F) point their origin to alkaline fluids. Type Ⅲ ferrocolumbite is featured by intergrowth with aeschynite within biotite-apatite veins that weakly altered the host dolomite. The coarse-grained dolomite (∼70 vol.%) has occasionally been cut by biotite-apatite veins (∼15 vol.%, Fig. 2G–I). Type Ⅲ ferrocolumbite (∼2 vol.%, 10–200 μm) is distributed within these veins and intimately associated with aeschynite. It shows irregular shapes and contains inclusions of biotite, apatite, and pyrite (Fig. 2H and I).

Representative thin sections were selected for further analysis (e.g. SEM and EPMA), with the targeted grains being drilled to make mounts for SIMS U-Pb dating. Detailed deposit geology, sample location, methods, and data results are presented in the Supplementary Data.

## RESULTS

**Figure 3.**
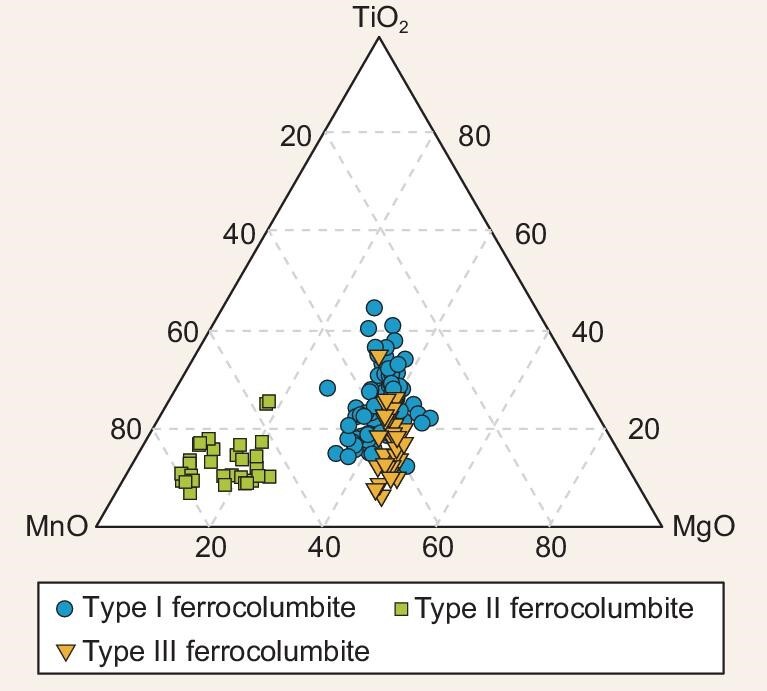
Ternary plot displaying the molar fractions of MnO, MgO, and TiO_2_ in three types of ferrocolumbite. Three types of ferrocolumbite are discerned through their mineral occurrences and paragenetic mineral associations of hydrothermal alteration in the hosted dolomite from the West orebody, Bayan Obo.

**Figure 4.**
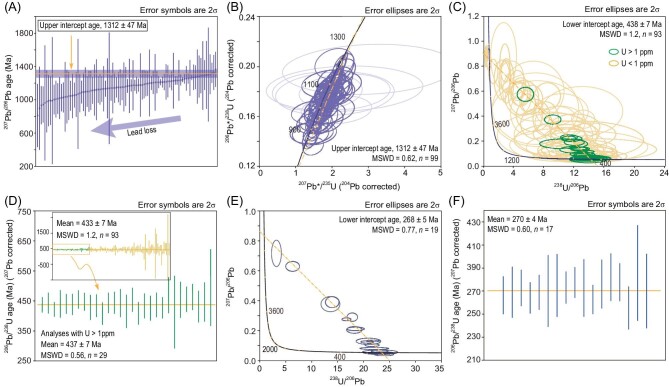
(A, B) ^207^Pb/^206^Pb ages and Wetherill Concordia plot of type I ferrocolumbite, six analyses in (B), represented by dull lilac ellipses, are excluded from (A) due to their unusually large ^207^Pb/^206^Pb age uncertainties. (C, D) Tera-Wasserburg plot and weighted average ^206^ Pb/^238^U age of type II ferrocolumbite. (E, F) Tera-Wasserburg plot and weighted average ^206^ Pb/^238^U age of type III ferrocolumbite. MSWD denotes mean square of weighted deviates.

All ferrocolumbites are Nb-enriched (Nb_2_O_5_ ≥ 76 wt%) and Ta-deficient (Ta_2_O_5_ ≤ 0.1 wt%; Set S1). Type Ⅰ and Ⅲ ferrocolumbites have comparable chemical compositions, with overlapping ranges of MnO contents (2.37 ± 1.00 wt%), MgO contents (1.41 ± 0.54 wt%), and TiO_2_ contents (1.65 ± 0.91 wt%), while type Ⅱ ferrocolumbite has higher MnO contents (4.72 ± 2.57 wt%), and lower MgO contents (0.57 ± 0.09 wt%) and TiO_2_ contents (0.98 ± 0.76 wt%; Fig. 3, Set S1). For type Ⅰ ferrocolumbite, 99 analyses were conducted on 71 grains. Their U concentrations are 29–550 ppm, with apparent ^207^Pb/^206^Pb dates ranging from 810 to 1366 Ma (Fig. 4A). After ^204^Pb-based common Pb correction, the 99 analyses defined an upper intercept age of 1312 ± 47 Ma (MSWD = 0.62, Fig. 4B) on the Wetherill Concordia plot. For type Ⅱ ferrocolumbite, 93 analyses were performed on 30 grains. Their U contents are very low (0.04–9.50 ppm). The 93 analyses defined a lower intercept age of 438 ±7 Ma (MSWD = 1.2) on the Tera-Wasserburg plot (Fig. 4C). The analyses of grains with U contents higher than 1 ppm show smaller uncertainties of ^206^ Pb/^238^U ages than those with U contents lower than 1 ppm (Fig. 4D). Twenty-nine analyses with U >1 ppm yield a weighted average ^206^ Pb/^238^U age of 437 ± 7 Ma (MSWD = 0.56, Fig. 4D) after common lead correction. For type Ⅲ ferrocolumbite, 19 analyses on 12 grains show U contents of 1.5–60 ppm. A lower intercept age of 268 ±5 Ma (MSWD = 0.77; Fig. 4E) was established on the Tera-Wasserburg plot, while a weighted average ^206^ Pb/^238^U age of 270 ± 4 Ma (MSWD = 0.60, Fig. 4F) after common lead correction was determined.

## DISCUSSION

### Three-stage Nb mineralization at Bayan Obo

Dating ore minerals directly is one of the best approaches to define the timing of mineralization (e.g. [30,31]), as this can place mineralizing mechanisms under a robust geological framework. Using SIMS U-Pb dating with a robust matrix-effect correction approach, we have successfully provided accurate U-Pb ages of ferrocolumbite, hence the age of Nb mineralization at Bayan Obo. In comparison to Th-Pb and Sm-Nd isochron ages, the dual decay system of U-Pb provides information on open system behaviors and incorporation of common lead [32], which is fundamental for robust interpretation of radiometric dates. For type Ⅰ ferrocolumbite, although many analyses exhibit significant uncertainties arising from low U and high levels of common lead contents, it clearly defines an array on the Wetherill Concordia plot (Fig. 4B) which is a testament to gradual lead loss (Fig. 4A). The oldest ^207^Pb/^206^Pb age (1366 ± 106 Ma), which experienced the least Pb loss, provides the closest estimate to the true age, and is consistent with the upper intercept age of 1312 ± 47 Ma within uncertainties (Fig. 4B). Therefore, the first-stage Nb mineralization was formed at ∼1.3 Ga. The exact timing of isotopic disturbances cannot be precisely defined due to considerable uncertainties in the lower intercept, but it is likely related to the second and third stage of Nb mineralization as discussed below.

For type Ⅱ ferrocolumbite, despite the fact that dates of low-U samples yield dates with significant uncertainties, high-U samples are more precise and are less impacted by common lead. High U samples give a weighted average ^206^Pb/^238^U age of 437 ± 7 Ma (*n* = 29, Fig. 4D), which is consistent with the lower intercept age of 438 ± 7 Ma defined by all spots (both high-U and low-U, *n* = 93, Fig. 4C). This 0.44 Ga age is supported by a Th-Pb isochron age of 438 ± 25 Ma [5] for aeschynite from the vein-type ores that cut through the orebodies and H8 dolomite.

For type Ⅲ ferrocolumbite, the lower intercept age of 268 ± 5 Ma is in good agreement with the weighted average ^206^ Pb/^238^U age of 270 ± 4 Ma (Fig. 2E and F), which clearly suggests a third stage of Nb mineralization at ∼0.27 Ga. This is supported by an aeschynite Th-Pb isochron age of ∼273 Ma [2].

### Genesis of Nb mineralization at Bayan Obo

Petrographic examination reveals that the ∼1.3 Ga ferrocolumbites are closely associated with hydrothermal minerals [33] such as apatite, biotite, chlorite, monazite, pyrite, and minor molybdenite (Fig. 2A–C). Hence we tentatively link the ∼1.3 Ga ferrocolumbites to hydrothermal fluids. We emphasize that their coexistence with hydrothermal minerals can also be explained by post-formation disturbance, as evidenced by their dissolution texture (Fig. 2B, C) and lead-loss nature (Fig. 4A). A Mesoproterozoic carbonatite magma intrusion is further supported by the carbonatite dykes near the mining area [8], which has been proposed as a major driver for the extensive fluorine and fenite alteration around the carbonatite dykes [34]. Given the considerable carry capacity of carbonatite magma for Nb [35], the first stage Nb metal could be sourced from Mesoproterozoic carbonatite magma.

The ∼0.44 Ga ferrocolumbites coexist with minerals typical of hydrothermal metasomatism in carbonatite systems [5,14], such as Sr- and Ba-rich minerals (norsethite, strontianite, barytocalcite, and baryte) and alkaline minerals (biotite and riebeckite, Fig. 2D–F), hence the second-stage Nb mineralization was suggested as hydrothermal in origin. Notably, the ∼0.44 Ga ferrocolumbites show distinct compositional differences from the ∼1.3 Ga ferrocolumbites, with lower TiO_2_ and MgO contents, and higher MnO contents (Fig. 3). Thus, the hydrothermal fluids responsible for second-stage Nb mineralization likely are richer in Sr, Ba, Mn, and alkali compared to that of the ∼1.3 Ga Nb mineralization. The ∼0.44 Ga Nb mineralization is coeval with the early Paleozoic hydrothermal veins that cut through the orebodies and H8 dolomite [25,26], linking it closely to the early Paleozoic hydrothermal activity. These hydrothermal fluids are proposed to be released from the subducting slab [5,36] or originate from the remelting of Mesoproterozoic carbonatite induced by heat generated through Paleozoic plate subduction [37]. However, ongoing debates persist regarding the subduction dynamics, with some studies proposing southward subduction of the Paleo-Asian Oceanic (PAO) plate towards the North China Craton (NCC) [38], while others suggest a northward subduction of South Bainaimiao Ocean, a branch of the PAO located to the north of the NCC [39]. Additionally, there is speculation that the hydrothermal fluids might originate from an alkaline-carbonatite suite that does not crop out on a plutonic scale in the Bayan Obo area [2]. These differing opinions emphasize the urgent need for further research to elucidate the mechanisms of early Paleozoic hydrothermal activity and specific processes of Nb enrichment and mineralization.

The ∼0.27 Ga ferrocolumbites are intergrown with aeschynite within biotite veins which cut through dolomite. They also host inclusions of biotite, pyrite, and apatite (Fig. 2G–I), hence are interpreted as hydrothermal in origin. The third-stage Nb mineralization is contemporaneous with Permian granites [28]. The significantly lower Nb content (16–19 ppm, [40]) of Permian granites compared to carbonatite dikes implies a limited contribution of Nb resource from the granites. However, the similar composition of the ∼0.27 Ga and ∼1.3 Ga ferrocolumbites (Fig. 3), and their close proximity in space (∼50 m apart; Fig. 1B) suggest that the ∼0.27 Ga Nb mineralization likely resulted from the reactivation of the ∼1.3 Ga Nb mineralization, facilitated by the Permian granite intrusion. This highlights the important role of granite emplace in the formation of high grade Nb mineralization, which challenges the traditional model that the Permian granites do not contribute metal endowment at Bayan Obo [41]. While the known hydrothermal and metamorphic effects of Permian granites primarily influence the eastern and southern sides of the H8 dolomite (e.g. [5]), the finding of ∼0.27 Ga ferrocolumbite in the West orebody suggests a more extensive impact. This new finding could offer indicative guidance for Nb resource exploration and extraction efforts in the Bayan Obo deposit.

Experimental studies suggest that Nb can be mobile in alkalic and F-rich hydrothermal systems [42]. Moreover, the major mechanisms driving Nb enrichment and mineralization involve fluorination and alkaline metasomatism [15]. Thus, giving the occurrence of alkaline minerals such as biotite and riebeckite, we emphasize the significance of alkaline fluids in all three stages of Nb mineralization.

### Implications for REE and Fe mineralization at Bayan Obo

Previous studies have yielded a wide range of radiometric dates using the Sm-Nd and Th-Pb systems for REE-bearing minerals (∼1.4–0.26 Ga, e.g. [9]) at Bayan Obo. The large range of Th-Pb dates has been interpreted as a continuous and prolonged REE mineralization event [10], or multiple stages of REE mineralization [43], or even the modifications of existing REE mineralization by later thermal-hydrothermal events [9]. These dates carry considerable uncertainties arising from unaccounted common lead and potential open system behaviors, which are difficult to evaluate by Th-Pb dating using LA-ICP-MS. Combined with detailed petrographic observation and the robust U-Pb system, our study overcame the aforementioned challenges and established a three-stage model for Nb mineralization. Given the close spatial association between REE and Nb minerals (Fig. 2), it is possible that REE mineralization may have also formed through multistage processes [43].

## METHODS

Representative thin sections of all samples were coated with carbon for backscattered electron (BSE) imaging by TESCAN integrated mineral analyzer (TIMA) and major element analysis by electron probe microanalyzer (EPMA). After that, suitable regions ∼5 mm in diameter were drilled out using a micro-drill. The drilled chips as well as the corresponding ferrocolumbite standards were mounted in epoxy mounts for SIMS U-Pb dating. Detailed methods of TIMA mineral mapping and EPMA major element analysis are presented in the Supplementary Data.

### SIMS ferrocolumbite U-Pb dating

The SIMS U-Pb analyses were performed using a Cameca IMS-1280HR SIMS at the Institute of Geology and Geophysics, Chinese Academy of Sciences (IGGCAS). The analytical procedure for ferrocolumbite mineral dating is similar to that developed by [18], only a brief summary is described here. The O^2−^ primary ion beam was accelerated at ∼13 kV, with an intensity of ∼6 nA. The ellipsoidal spot is about 10 × 15 μm in size. The ^93^Nb_2_^16^O^+^ peak is used as a reference peak for centering the secondary ion beam, energy, and mass adjustments. A mass resolution of ∼13 000 (defined at 50% peak height) was used to separate isobaric interferences on the ^204^Pb isotope. A single electron multiplier was used in ion-counting mode to measure secondary-ion beam intensities by a peak jumping sequence, including isotopes of ^93^Nb_2_^16^O^+^, Pb^+^, Th^+^, U^+^, UO^+^, and ^238^U^16^O^2+^. Each measurement consisted of 7 cycles, and the total analysis time of a single spot was ∼16 minutes.

To estimate the Pb/U ages of the ferrocolumbite samples in the absence of a matrix-matched standard, the matrix-effect correction strategy recommended by [18] was applied. First, two columbite-tantalite reference materials (NP-02 and ZTA01) of variable Nb/Ta chemical composition have been used as standards. The recommended ages of NP-02 and ZTA01 are 380.3 ± 2.4 Ma [18] and 264 Ma [44], respectively. ^206^ Pb/^238^U calibration was done based on the linear relationship between ^238^U^16^O^+^/^238^U^+^ and ^206^Pb^+^/^238^U^+^ ratios. Then, the Nb/Ta chemical composition of the ferrocolumbite samples and the standards were measured by EPMA. Based on the linear correlation between Nb/Ta chemical composition and SIMS age bias, the SIMS matrix-effect can be properly corrected. A long-term uncertainty of 1.5% (1 RSD) for ^206^ Pb/^238^U measurements was propagated to the unknowns.

According to formulas (1–3) [32], the measured U-Pb isotopic compositions were corrected for common lead using non-radiogenic ^204^Pb for type Ⅰ ferrocolumbites (sample BK59F-5–7, BK59F-5–8, BK59F-5–15, and BK59F-5–17), employing the terrestrial lead isotope model [45]. A Tera-Wasserburg plot [46] was constructed with common lead uncorrected data to deduce the common lead composition for type Ⅱ and Ⅲ ferrocolumbites (sample EB21-PM and BK59F-4–11). Then, a ^207^Pb-based common lead correction method was conducted for a single analysis of type Ⅱ and Ⅲ ferrocolumbites.


(1) \begin{eqnarray*}
{{f}_{206}} = \frac{{\left( {{}_{}^{206}Pb/{}_{}^{204}Pb} \right)\textit{common}}}{{\left( {{}_{}^{206}Pb/{}_{}^{204}Pb} \right)\textit{measured}}},
\end{eqnarray*}

(2) \begin{eqnarray*}
\frac{{{}_{}^{206}Pb*}}{{{}_{}^{238}U}} = \frac{{{}_{}^{206}Pb\ \textit{measured}}}{{{}_{}^{238}U}} \times \left( {1 - {{f}_{206}}} \right),
\end{eqnarray*}

(3) \begin{eqnarray*}
&&\frac{{{}_{}^{207}Pb*}}{{{}_{}^{206}Pb*}} =\\
&&\frac{{\left( {{}_{}^{207}Pb/{}_{}^{206}Pb} \right)\textit{measured} - \left( {{}_{}^{207}Pb/{}_{}^{206}Pb} \right)\textit{common} \times {{f}_{206}}}}{{1 - {{f}_{206}}}}.\\
\end{eqnarray*}

Data reduction was carried out using the IsoplotR program [47]. Uncertainties on individual analyses in data tables are reported at the 1σ level. The final U-Pb age result is quoted with a 95% confidence interval.

To monitor the precision and accuracy of SIMS U-Pb ferrocolumbite in this study, two in-house columbite standards LCT01 and LCT02 were alternately analyzed as an unknown together with other unknown columbite samples. The independent ^207^Pb/^206^Pb ages of LCT01 and LCT02 are weighted at 1802 ± 5 Ma and 919 ± 4 Ma, respectively. With the above-mentioned calibration procedure, LCT01 and LCT02 yield weighted average ^206^ Pb/^238^U ages of 1808 ± 19 Ma and 918 ± 6 Ma, respectively, which are identical within error with their values (see Set S2). The results of in-house columbite standards indicate that our SIMS U-Pb columbite dating method is accurate.

## Supplementary Material

nwae063_Supplemental_Files

## References

[1] U.S. Geological Survey . Mineral Commodity Summaries 2023. https://www.usgs.gov/publications/mineral-commodity-summaries-2023 (11 March 2024, date last accessed).

[2] Drew LJ, Meng Q, Sun W. The Bayan Obo iron-rare-earth-niobium deposits, Inner Mongolia, China. Lithos 1990; 26: 43–65. 10.1016/0024-4937(90)90040-8

[3] Philpotts J, Tatsumoto M, Li X et al. Some Nd and Sr isotopic systematics for the REE-enriched deposit at Bayan Obo, China. Cheml Geol 1991; 90: 177–88. 10.1016/0009-2541(91)90098-C

[4] Wang J, Tatsumoto M, Li X et al. A precise ^232^Th-^208^Pb chronology of fine-grained monazite: age of the Bayan Obo REE-Fe-Nb ore deposit, China. Geochim Cosmochim Acta 1994; 58: 3155–69. 10.1016/0016-7037(94)90043-4

[5] Chao ECT, Black JM, Minkin JA et al. The Sedimentary Carbonate-hosted Giant Bayan Obo REE-Fe-Nb Ore Deposit of Inner Mongolia, China: a Cornerstone Example for Giant Polymetallic Ore Deposits of Hydrothermal Origin. Washington DC: US Government Printing Office, 1997.

[6] Ren YC, Zhang YC, Zhang ZQ. Study on heat events of ore-forming Bayan Obo deposit (in Chinese with English abstract). Acta Geoscientia Sinica 1994; 15: 95–101.

[7] Li Q, Liu Y, Tang G et al. Zircon Th–Pb dating by secondary ion mass spectrometry. J Anal Atom Spectrom 2018; 33: 1536–44. 10.1039/C8JA00125A

[8] Fan H-R, Hu F-F, Yang KF et al. Integrated U–Pb and Sm–Nd geochronology for a REE-rich carbonatite dyke at the giant Bayan Obo REE deposit, Northern China. Ore Geol Rev 2014; 63: 510–9. 10.1016/j.oregeorev.2014.03.005

[9] Li X-C, Yang K-F, Spandler C et al. The effect of fluid-aided modification on the Sm-Nd and Th-Pb geochronology of monazite and bastnäsite: implication for resolving complex isotopic age data in REE ore systems. Geochim Cosmochim Acta 2021; 300: 1–24. 10.1016/j.gca.2021.02.028

[10] Song W, Xu C, Smith MP et al. Genesis of the world's largest rare earth element deposit, Bayan Obo, China: protracted mineralization evolution over ∼1 b.y. Geology 2018; 46: 323–6. 10.1130/G39801.1

[11] Institute of Geochemistry, Chinese Academy of Sciences . Geochemistry of Bayan Obo Ore Deposit (in Chinese). Beijing: Science Press, 1988.

[12] Liu S, Ding L, Fan H-R et al. Hydrothermal genesis of Nb mineralization in the giant Bayan Obo REE-Nb-Fe deposit (China): implicated by petrography and geochemistry of Nb-bearing minerals. Precambrian Res 2020; 348: 105864. 10.1016/j.precamres.2020.105864

[13] Wendt I, Carl C. The statistical distribution of the mean squared weighted deviation. Chem Geol 1991; 86: 275–85. 10.1016/0168-9622(91)90010-T

[14] Smith MP, Campbell LS, Kynicky J. A review of the genesis of the world class Bayan Obo Fe–REE–Nb deposits, Inner Mongolia, China: multistage processes and outstanding questions. Ore Geol Rev 2015; 64: 459–76. 10.1016/j.oregeorev.2014.03.007

[15] Ren Y, Yang X, Yang X et al. Mineralogical study on the distribution regularity of niobium in various types of ores in the giant Bayan Obo Fe-REE-Nb deposit. Ore Geol Rev 2023; 161: 105602. 10.1016/j.oregeorev.2023.105602

[16] Zhang Q . Analysis of the basic mineralogical characteristics of niobium resources in the Bayan Ebo deposit (in Chinese). Nonferrous Metals 2005; 57: 111–3.

[17] Cheng MQ . Characteristics of niobium minerals and feasibility of niobium utilization at Bayan Ebo Nb deposit (in Chinese with English abstract). In: 1997 China Iron and Steel Annual Conference. 1997, p. 19–22.

[18] Legros H, Mercadier J, Villeneuve J et al. U-Pb isotopic dating of columbite-tantalite minerals: development of reference materials and in situ applications by ion microprobe. Cheml Geol 2019; 512: 69–84. 10.1016/j.chemgeo.2019.03.001

[19] Yang X-M, Le Bas MJ. Chemical compositions of carbonate minerals from Bayan Obo, Inner Mongolia, China: implications for petrogenesis. Lithos 2004; 72: 97–116. 10.1016/j.lithos.2003.09.002

[20] Yang K, Fan H, Pirajno F et al. The Bayan Obo (China) giant REE accumulation conundrum elucidated by intense magmatic differentiation of carbonatite. Geology 2019; 47: 1198–202. 10.1130/G46674.1

[21] Campbell LS, Compston W, Sircombe KN et al. Zircon from the East Orebody of the Bayan Obo Fe–Nb–REE deposit, China, and SHRIMP ages for carbonatite-related magmatism and REE mineralization events. Contrib Mineral Petrol 2014; 168: 1041. 10.1007/s00410-014-1041-3

[22] Zhang S-H, Zhao Y, Liu Y. A precise zircon Th-Pb age of carbonatite sills from the world's largest Bayan Obo deposit: implications for timing and genesis of REE-Nb mineralization. Precambrian Res 2017; 291: 202–19. 10.1016/j.precamres.2017.01.024

[23] Li X-C, Fan H-R, Zeng X et al. Identification of ∼1.3Ga hydrothermal zircon from the giant Bayan Obo REE deposit (China): implication for dating geologically-complicated REE ore system. Ore Geol Rev 2021; 138: 104405. 10.1016/j.oregeorev.2021.104405

[24] Le Bas MJ, Keller J, Kejie T et al. Carbonatite dykes at Bayan Obo, Inner Mongolia, China. Miner Petrol 1992; 46: 195–228. 10.1007/BF01164647

[25] Hu F-F, Fan H-R, Liu S et al. Samarium-neodymium and rubidium-strontium isotopic dating of veined REE mineralization for the Bayan Obo REE-Nb-Fe Deposit, Northern China. Resour Geol 2009; 59: 407–14. 10.1111/j.1751-3928.2009.00107.x

[26] Liu YL, Yang G, Chen JF et al. Re-Os dating of pyrite from giant Bayan Obo REE-Nb-Fe deposit. Chin Sci Bull 2004; 49: 2627–31. 10.1360/04wd0185

[27] Zhang ZQ, Tang SH, Wang JH et al. New data for ore-forming age of the Bayan Obo REE deposit, Inner Mongolia (in Chinese with English abstract). Acta Geoscientia Sinica 1994; 1–2: 85–94.

[28] Ling M-X, Zhang H, Li H et al. The permian–Triassic granitoids in Bayan Obo, North China Craton: a geochemical and geochronological study. Lithos 2014; 190–191: 430–9. 10.1016/j.lithos.2014.01.002

[29] Zhang S-H, Zhao Y, Li Q-L et al. First identification of baddeleyite related/linked to contact metamorphism from carbonatites in the world's largest REE deposit, Bayan Obo in North China Craton. Lithos 2017; 284–285: 654–65. 10.1016/j.lithos.2017.05.015

[30] Li Y, Selby D, Condon D et al. Cyclic magmatic-hydrothermal evolution in porphyry systems: high-precision U-Pb and re-Os geochronology constraints on the Tibetan Qulong porphyry Cu-Mo deposit. Econ Geol 2017; 112: 1419–40. 10.5382/econgeo.2017.4515

[31] Saintilan NJ, Selby D, Creaser RA et al. Sulphide re-Os geochronology links orogenesis, salt and Cu-Co ores in the Central African Copperbelt. Sci Rep 2018; 8: 14946. 10.1038/s41598-018-33399-7 30297732 PMC6175924

[32] Williams IS . U-Th-Pb geochronology by ion microprobe. In: Mckibben MA, Shanks WC III, Ridley WI (eds.). Applications of Microanalytical Techniques to Understanding Mineralizing Processes. Littleton: Society of Economic Geologists 1998.

[33] Liu S, Fan H-R, Yang K-F et al. Fenitization in the giant Bayan Obo REE-Nb-Fe deposit: implication for REE mineralization. Ore Geol Rev 2018; 94**:** 290–309. 10.1016/j.oregeorev.2018.02.006

[34] Liu S, Fan H-R, Yang K-F et al. Mesoproterozoic and paleozoic hydrothermal metasomatism in the giant Bayan Obo REE-Nb-Fe deposit: constrains from trace elements and Sr-Nd isotope of fluorite and preliminary thermodynamic calculation. Precambrian Res 2018; 311: 228–46. 10.1016/j.precamres.2018.04.021

[35] Mitchell RH . Primary and secondary niobium mineral deposits associated with carbonatites. Ore Geol Rev 2015; 64: 626–41. 10.1016/j.oregeorev.2014.03.010

[36] Ling MX, Liu YL, Williams IS et al. Formation of the world's largest REE deposit through protracted fluxing of carbonatite by subduction-derived fluids. Sci Rep 2013; 3: 1776.

[37] She H-D, Fan H-R, Santosh M et al. Paleozoic remelting of carbonatite in Bayan Obo (China): further insights into the formation of a giant REE deposit. Gondwana Res 2023; 119: 172–85. 10.1016/j.gr.2023.03.018

[38] Xiao W, Windley BF, Hao J et al. Accretion leading to collision and the Permian Solonker suture, Inner Mongolia, China: termination of the central Asian orogenic belt. Tectonics 2003; 22: 1069. 10.1029/2002TC001484

[39] Zeng H, Song D, Xiao W et al. Field geology and provenance analyses of the Ganqimaodu accretionary complex (Inner Mongolia, China): implications for early paleozoic tectonic evolution of the southern Central Asian Orogenic Belt. Int J Earth Sci 2022; 111: 2633–56. 10.1007/s00531-021-02148-z

[40] Zhang ZQ, Yuan ZX, Tang SH et al. Age and Geochemistry of the Bayan Obo Ore Deposit (in Chinese). Beijing: Geological Publishing House, 2003.

[41] Fan H-R, Hu F-F, Yang KF et al. Geochronology framework of late paleozoic dioritic-granitic plutons in the Bayan Obo area, Inner Mongolia, and tectonic significance (in Chinese with English abstract). Acta Petrol Sin 2009; 25: 2933–8.

[42] Akinfiev NN, Korzhinskaya VS, Kotova NP et al. Niobium and tantalum in hydrothermal fluids: thermodynamic description of hydroxide and hydroxofluoride complexes. Geochim Cosmochim Acta 2020; 280: 102–15. 10.1016/j.gca.2020.04.009

[43] Yang X, Lai X, Pirajno F et al. Genesis of the Bayan Obo Fe-REE-Nb formation in Inner Mongolia, North China Craton: a perspective review. Precambrian Res 2017; 288: 39–71. 10.1016/j.precamres.2016.11.008

[44] Zhao JX, He CT, Qin KZ et al. Geochronology, source features and the characteristics of fractional crystallization in pegmatite at the Qongjiagang giant pegmatite-type lithium deposit, Himalaya, Tibet (in Chinese with English abstract). Acta Petrol Sin 2021; 37: 3325–47.

[45] Stacey J, Kramers J. Approximation of terrestrial lead isotope evolution by a two-stage model. Earth Planet Sci Lett 1975; 26: 207–21. 10.1016/0012-821X(75)90088-6

[46] Tera F, Wasserburg GJ. U-Th-Pb systematics in three Apollo 14 basalts and the problem of initial Pb in lunar rocks. Earth Planet Sci Lett 1972; 14: 281–304. 10.1016/0012-821X(72)90128-8

[47] Vermeesch P . IsoplotR: a free and open toolbox for geochronology. Geosci Front 2018; 9: 1479–93. 10.1016/j.gsf.2018.04.001

[48] Zhang ZQ, Tang SH, Yuan ZX et al. The Sm-Nd and Rb-Sr isotope systems of the dolomites in the Bayan Obo ore deposit, Inner Mongolia, China (in Chinese with English abstract). Acta Petrol Sin 2001; 17: 637–42.

[49] Zhang PS, Tao KJ. Bayan Obo Mineralogy (in Chinese). Beijing: Science Press, 1986.

