# Supplementary material for: Three-stage niobium mineralization at Bayan Obo, China
Source: Natl Sci Rev. 2024 Feb 22;11(4):nwae063. doi: 10.1093/nsr/nwae063 (PMC11018119; doi:10.1093/nsr/nwae063)
Supplement: nwae063_Supplemental_Files [file nwae063_supplemental_files.zip › Supplementary data.docx]

**Supplementary data for**

**Three-stage niobium mineralization at Bayan Obo, China**

**Yan Yu^1, 2^, Yang Li^3*^, Yu Liu^1^, Xiao-Xiao Ling^1^, Li-Guang Wu^1^, Li Yang^4^, Lan Yang^1, 2^, Bo Yang^1, 2, 4^, Yong-Gang Zhao^4^, Xian-Hua Li^1, 2, 5*^**

^1^State Key Laboratory of Lithospheric Evolution, Institute of Geology and Geophysics, Chinese Academy of Sciences, Beijing 10029, China

^2^College of Earth and Planetary Science, University of Chinese Academy of Science, Beijing 100049, China

^3^Ministry of Education Key Laboratory of Orogenic Belts and Crustal Evolution, School of Earth and Space Sciences, Peking University, Beijing 100871, China

^4^Mining Research Institute, Baotou Iron and Steel (Group) Co., LTD, Baotou 014010, China

^5^State Key Laboratory of Baiyunobo Rare Earth Resource Research and Comprehensive Utilization, Baotou 014000, China

*Corresponding authors: [geoliy@outlook.com](mailto:geoliy@outlook.com); [lixh@gig.ac.cn](mailto:lixh@gig.ac.cn);

**1. Deposit Geology**

The Bayan Obo ore deposit is located on the northern margin of the North China Craton (NCC) in Inner Mongolia, China. The oldest basement rock at Bayan Obo consists of Neoarchean (~2.5 Ga) and Paleoproterozoic (~2.0 to ~1.9 Ga) migmatite [1,2]. Subsequently, the NCC underwent a period of rifting during the breakup of the Columbia supercontinent [3, 4], which developed a thick sequence of sediments (i.e., Bayan Obo Group).

The Bayan Obo Group is dominated by sandstone, slate, and limestone. It has been subdivided into nine lithologic units (H1–H9 units, [5]). The H8 unit is composed of dolomitic marble and is distributed along the west-east with a length of ~18 km and a width of up to 3 km (Fig. 1A, [6,7]). It hosts the vast majority of the ore deposit (Fig. 1A). The Bayan Obo deposit is currently composed of three main ore bodies, the West, Main, and East orebody (Fig. 1A), which predominantly host Fe, REE, and Nb resources. The Main and East orebody each consists of a single lenticular-shaped orebody, while the West orebody is composed of several small orebodies (Fig. 1B, [5,8]). The principal REE-bearing minerals are monazite and REE fluorocarbonates, the main Fe-bearing minerals are magnetite and hematite, and the major Nb-bearing minerals are columbite, ilmenorutile, pyrochlore, and aeschynite. Aeschynite is the most common Nb-bearing mineral in the Main and East orebody, although pyrochlore, columbite, and ilmenorutile are also found [9,10]. Niobium mineralization was dominantly found in the West orebody and mainly hosted by columbite, subordinately hosted by aeschynite, fersmite, pyrochlore, and baotite [6,10,11]. Previous studies suggested that the columbite accounts for up to ~90% Nb budget in the dolomite host rock and up to 80% in the Nb-REE-Fe ore [10,12].

Abundant carbonatite dykes (1–2 m thick) have been recognized adjacent to the ore deposit [13,14]. They intruded into the Archean basement and cut through the Bayan Obo Group, producing intense fenitization of the surrounding wall rocks [1]. Zircons from these dykes have a SIMS Th-Pb peak age of 1327 ± 20 Ma [15] indicating that carbonatite dykes were intruded at ~1.3 Ga. It was reported that zircon cores from the East orebody having a SHRIMP Th-Pb age of 1325 ± 60 Ma [7], and zircons from the H8 dolomite and East orebody having LA-ICP-MS weighted Th-Pb ages of 1301 ± 12 Ma [16] and 1297 ± 13 Ma [17], respectively. The similar geochemical characteristics between the H8 dolomite and the carbonatite dikes further suggest their close genetic association [18,19].

Undeformed to weakly deformed veins cut through the orebodies and H8 dolomite [20,21]. These vein-like ores are diverse in composition, including gangue minerals of fluorite, aegirine, alkaline amphibole, mica, pyrite, baryte, and molybdenite, indicating intense hydrothermal activity. The hydrothermal activity has been confirmed to occur at the early Paleozoic through various dating methods, including Sm-Nd isochron ages of 422 ± 18 Ma for huanghiote, albite and riebeckite [21] and 442 ± 42 Ma for huanghoite, aegirine, fluorite and calcite [20], a Rb-Sr isochron age of 459 ± 41 Ma for biotite [20], a Re-Os isochron age of 439 ± 86 Ma for pyrite [22], and a Th-Pb peak age of 455 ± 28 Ma for the metamorphic or hydrothermal rims of zircon [7].

The ore deposit was also affected by abundant Permian granites which were observed to the south and east of the deposit (Fig. 1A, [20,23]). These granites have zircon U-Pb ages of ~240–280 Ma [24,25] and produce the biotite-rich skarn at contact with the H8 dolomite [26,27]. The presence of magnesian skarn minerals (e.g., humite, diopside, and phlogopite) in the baddeleyite that has an average ^207^Pb/^206^Pb age of 279 ± 10 Ma provides evidence of hydrothermal and contact metamorphic processes occurring between the carbonatite and the Permian granitoid intrusion [27].

**2. Sample Location**

This study selected six dolomite samples with three types of columbites in the West orebody (Fig. 1B) for SIMS columbite dating. Four samples (BK59F-5-7, BK59F-5-8, BK59F-5-15, and BK59F-5-17) were collected from the BK59F-5 drill hole (N41°48′06.1″, E109°54′38.5″). They were obtained from depths of 65.4 m, 65.8 m, 76.1 m, and 84.6 m, respectively, and contain type Ⅰ columbites. Sample EB21-PM-24 was collected from the 1587 m outcrop (N41°48′03″, E109°50′46″) in the western part of the West Mining pit and contains type Ⅱ columbites. Sample BK59F-4-11 was from a depth of 146.3 m in the BK59F-4 drill hole (N41°48′07.7″, E109°54′38.3″), and contains type Ⅲ columbites.

**3. Methods**

**3.1 TIMA mineral mapping**

Mineral mapping was performed on carbon-coated thin sections using a TESCAN TIMA system which is equipped with a MIRA3 scanning electron microscope at Guangzhou Tuoyan Testing Technology Company. The analyses used an accelerable voltage of 25 kV and a probe current of 8.24 nA. The working distance was set to 15 mm. Pixel spacing was set to 2 μm and dot spacing was set to 6 μm. The current and BSE signal intensity was automatically calibrated on a platinum Faraday cup. Energy Dispersive Spectroscopy (EDS) was checked using a manganese standard. The TIMA liberation analysis module was used in the test.

**3.2 EPMA major element analysis**

Elemental compositions of columbite were determined using a CAMECA SXFive EPMA in conjunction with thallium acid phthalate (TAP), and large LiF and polyethylene terephthalate (PET) diffraction crystals at the Institute of Geology and Geophysics, Chinese Academy of Sciences (IGGCAS). The accelerating voltage was 20 kV, the beam size was ~5 μm, and the beam current was 50 nA. The peak counting time for each element was ca. 10–20 s, and a single spot's total acquisition time was ca. 5 min. For the calibration procedure, a natural rhodonite is used as the Mn standard, a natural specularite for Fe, a synthetic periclase for Mg, a synthetic pretulite for Sc, two synthetic metals for Nb and Ta, and a synthetic rutile for Ti. Matrix compositional effects were corrected using the CAMECA software X-PHI. Detailed instrumental conditions are provided in Supplementary Data Set S1.

**4. Columbite U-Pb dating results**

A total of 99 analyses were performed on 71 individual columbite grains from the type Ⅰ sample. These columbite grains contain 29–550 ppm U and 1–93 ppm Th, with a Th/U ratio of 0.03–0.41. They have variable common lead compositions (expressed as f_206_, which is the ratio of common ^206^Pb to total ^206^Pb) ranging from 1.8% to 33.5%. After ^204^Pb-based common Pb correction, these 99 analyses define an upper intercept age of 1312 ± 47 Ma (2σ, MSWD [mean squared weighted deviation] = 0.62) on the Wetherill Concordia plot (Fig. 4A). Six analyses (numbered as BK59F-5-7@50-8, BK59F-5-7@60-2, BK59F-5-15@70, BK59F-5-15@71, BK59F-5-15@72-1, and BK59F-5-15@72-2, Supplementary Data Set S2) have abnormally large ^207^Pb/^206^Pb age uncertainties (1σ > 400 Ma, dull lilac symbols in Fig. 4A) due to the lower U contents and/or higher common lead compositions. Excluding the above 6 analyses, the rest 93 analyses yield apparent ^207^Pb/^206^Pb ages between 810 and 1366 Ma (Fig. 4B).

A total of 93 analyses were conducted on 30 individual columbite grains from the type Ⅱ sample. They have low U (0.04–9.50 ppm) and Th contents (0.59–250 ppm) with Th/U ratios of 1.6–2889.0. The f_206_ values vary from 0% to 100%. The 93 analyses define a lower intercept age of 438 ± 7 Ma (2σ, MSWD = 1.2) on the Tera-Wasserburg plot (Fig. 4 C). The ^207^Pb-corrected ^206^Pb/^238^U ages yield a weighted average of 433 ± 7 Ma (2σ, MSWD = 1.2, n = 93; Fig. 4 D inset). The spots of higher U contents (> 1 ppm) show smaller uncertainties of ^206^Pb/^238^U ages than those with U contents lower than 1 ppm (Fig. 4 D). Twenty-nine analyses with U > 1 ppm yield a weighted average ^207^Pb-corrected ^206^Pb/^238^U age of 437 ±7 Ma (2σ, MSWD = 0.56, Fig. 4D). Three analyses (numbered as EB21-PM-24@20-1, EB21-PM-24@20-2, and EB21-PM-24@21-6, Supplementary Data Set S2) have abnormally large ^206^Pb/^238^U age uncertainties (1σ > 570 Ma) due to high f_206_ values (> 93%).

Nineteen analyses were performed on 12 individual columbite grains from type Ⅲ sample. They yield U contents of 1.5–60 ppm, Th contents of 2–189 ppm, and Th/U ratios of 0.4–5.6. They have variable common lead compositions with f_206_ ranging between 0% and 91%. The 19 analyses yield a lower intercept age of 268 ± 5 Ma (2σ, MSWD = 0.77; Fig. 4 E) on the Tera-Wasserburg plot. Two spots (numbered as BK59F-4-11@9 and BK59F-4-11@12-2, Supplementary Data Set S2) have large ^206^Pb/^238^U age uncertainties (1σ > 45 Ma) due to the high common lead compositions (f_206_ = 91% and 82%) and low U concentrations (2.6 ppm and 5.3 ppm). Excluding the above two analyses, the rest 17 analyses yield a weighted average ^207^Pb-corrected ^206^Pb/^238^U age of 270 ± 4 Ma (2σ, MSWD = 0.60) (Fig. 4 F).

**List of Data Sets**

**Supplementary Data Set S1.** Major element composition of three types of columbite.

**Supplementary Data Set S2.** SIMS U-Pb dating of columbite.

**References**

1. Fan HR, Yang KF and Hu FF *et al.* The giant Bayan Obo REE-Nb-Fe deposit, China: Controversy and ore genesis. *Geosci Front* 2016; **7**: 335–44.

2. Fan HR, Yang KF and Hu FF *et al.* Zircon geochronology of basement rocks from the Bayan Obo area, Inner Mongolia, and tectonic implications (in Chinese). *Acta Petrol Sin* 2010; **26**: 1342–50.

3. Zhao GC, Sun M and Wilde SA *et al.* Assembly, Accretion and Breakup of the Paleo-Mesoproterozoic Columbia Supercontinent: Records in the North China Craton. *Gondwana Res* 2003; **6**: 417–34.

4. Zhao GC, Li SZ and Sun M *et al.* Assembly, accretion, and break-up of the Palaeo-Mesoproterozoic Columbia supercontinent: record in the North China Craton revisited. *Int Geol Rev* 2011; **53**: 1331–56.

5. Chao ECT, Back JM and Minkin JA *et al.* Host-rock controlled epigenetic, hydrothermal metasomatic origin of the Bayan Obo REE-Fe-Nb ore deposit, Inner Mongolia, P.R.C. . *Appl Geochem* 1992; **7**: 443–58.

6. Chao ECT, Black JM, Minkin JA *et al.* *The sedimentary carbonate-hosted giant Bayan Obo REE-Fe-Nb ore deposit of Inner Mongolia, China: a cornerstone example for giant polymetallic ore deposits of hydrothermal origin*: US Government Printing Office, 1997.

7. Campbell LS, Compston W and Sircombe KN *et al.* Zircon from the East Orebody of the Bayan Obo Fe–Nb–REE deposit, China, and SHRIMP ages for carbonatite-related magmatism and REE mineralization events. *Contrib Mineral Petr* 2014; **168**: 1–23.

8. Drew LJ, Meng QR and Sun WJ. The Bayan Obo iron-rare-earth-niobium deposits, Inner Mongolia, China. *Lithos* 1990; **26**: 43–65.

9. Tao KJ, Yang ZM and Zhang PS. Study on the occurrence of rare earth and niobium minerals in the Baiyun Ebo mining area (in Chinese). In: *Proceedings of the Fourth Academic Conference of the Chinese Society of Rare Earths,* *2000*.

10. Zhang QF. Analysis of the basic mineralogical characteristics of niobium resources in the Bayan Ebo deposit (in Chinese). *Nonferrous Metals* 2005; **57**: 111–3.

11. Smith MP and John S. The chemistry of niobium mineralisation at Bayan Obo, Inner Mongolia, China: constraints on the hydrothermal precipitation and alteration of Nb-minerals. *Acta Geol Sin- Engl* 2012; **86**: 700–22.

12. Cheng MQ. Characteristics of niobium minerals and feasibility of niobium utilization at Bayan Ebo Nb deposit (in Chinese). In: *1997 China Iron and Steel Annual Conference,* *1997*, p. 19–22.

13. Fan HR, Hu FF and Yang KF *et al.* Integrated U–Pb and Sm–Nd geochronology for a REE-rich carbonatite dyke at the giant Bayan Obo REE deposit, Northern China. *Ore Geol Rev* 2014; **63**: 510–19.

14. Le Bas MJ, Keller J and Kejie T *et al.* Carbonatite dykes at bayan Obo, inner Mongolia, China. *Miner Petrol* 1992; **46**: 195–228.

15. Li QL, Liu Y and Tang GQ *et al.* Zircon Th–Pb dating by secondary ion mass spectrometry. *J Anal Atom Spectrom* 2018; **33**: 1536–44.

16. Zhang SH, Zhao Y, Liu YS. A precise zircon Th-Pb age of carbonatite sills from the world’s largest Bayan Obo deposit: Implications for timing and genesis of REE-Nb mineralization. *Precambrian Res* 2017; **291**: 202–19.

17. Li XC, Fan HR and Zeng X *et al.* Identification of ~1.3Ga hydrothermal zircon from the giant Bayan Obo REE deposit (China): Implication for dating geologically-complicated REE ore system. *Ore Geol Rev* 2021; **138**: 104405.

18. Yang KF, Fan HR and Santosh M *et al.* Mesoproterozoic carbonatitic magmatism in the Bayan Obo deposit, Inner Mongolia, North China: Constraints for the mechanism of super accumulation of rare earth elements. *Ore Geol Rev* 2011; **40**: 122–31.

19. Yang KF, Fan HR and Pirajno F *et al.* The Bayan Obo (China) giant REE accumulation conundrum elucidated by intense magmatic differentiation of carbonatite. *Geology* 2019; **47**: 1198–202.

20. Hu FF, Fan HR and Liu S *et al.* Samarium-Neodymium and Rubidium-Strontium Isotopic Dating of Veined REE Mineralization for the Bayan Obo REE-Nb-Fe Deposit, Northern China. *Resour Geol* 2009; **59**: 407–14.

21. Zhang ZQ, Tang SH and Wang JH *et al.* New data for ore-forming age of the Bayan Obo REE deposit, Inner Mongolia (in Chinese). *Acta Geoscientia Sinica* 1994; **1-2**: 85–94

22. Liu YL, Yang G and Chen JF *et al.* Re-Os dating of pyrite from Giant Bayan Obo REE-Nb-Fe deposit. *Chinese Sci Bull* 2004; **49**: 2627–31.

23. Yang XM, Yang XY and Fan HR *et al.* Rare earth elements geochemistry of the Hercynian granitic complex in Bayan Obo (in Chinese). *Chinese Rare Earths* 2000; **2**: 1–7.

24. Fan HR, Hu FF and Yang KF *et al.* Geochronology framework of late Paleozoic dioritic-granitic plutons in the Bayan Obo area, Inner Mongolia, and tectonic significance.(in Chinese). *Acta Petrol Sin* 2009; **25**: 2933–38.

25. Ling MX, Zhang H and Li H *et al.* The Permian–Triassic granitoids in Bayan Obo, North China Craton: A geochemical and geochronological study. *Lithos* 2014; **190–191**: 430–9.

26. Yang KF, Fan HR, Hu FF *et al.* The age of skarn mineralization in the giant REE-Nb-Fe mining area of Bayan Obo: the Rb-Sr legal year of single particle metallomica.(in Chinese). *Acta Petrol Sin* 2007; **5**: 1018–22.

27. Zhang SH, Zhao Y and Li QL *et al.* First identification of baddeleyite related/linked to contact metamorphism from carbonatites in the world's largest REE deposit, Bayan Obo in North China Craton. *Lithos* 2017; **284–285**: 654–65.
